# Fatigue prediction needs time: comparing the diagnostic value of short vs. prolonged cognitive load

**DOI:** 10.1007/s00415-025-13510-5

**Published:** 2025-11-13

**Authors:** Stefanie Linnhoff, Tino Zaehle

**Affiliations:** 1 Department of Neurology, Otto-von-Guericke University, Magdeburg, Germany; 2 Center for Behavioral Brain Sciences, Otto-von-Guericke University, Magdeburg, Germany; 3 Institute for Medical Psychology, Otto-von-Guericke University, Magdeburg, Germany; 4 German Centre for Mental Health (DZPG), partner site Halle-Jena-Magdeburg, Magdeburg, Germany; 5 University Clinic Magdeburg, Leipziger Street 44, 39120 Magdeburg, Germany

Dear Sirs,

Fatigue represents one of the most challenging symptoms to address in neurological disorders, particularly in multiple sclerosis (MS), where it affects up to 80% of people with MS (pwMS) contributing substantially to reduced daily functioning, work capacity, and socio-economic burden [1, 2]. Clinically, fatigue can be divided into subjective fatigue, the self-perceived sense of exhaustion and objective fatigability, referring to measurable declines in cognitive or motor performance over time [3, 4]. While subjective fatigue is typically assessed through self-report questionnaires, these tools are inherently vulnerable to recall bias and individual interpretation [5], underscoring the need for objective markers.

Prolonged sustained-attention paradigms, particularly Continuous Performance Tasks (CPTs), which require maintaining vigilance for 20–30 min or longer, have consistently proven sensitive for detecting cognitive fatigability in MS, reliably revealing temporal performance decline that correlates with self-reported fatigue [4, 6, 7]. However, their clinical utility is limited by long administration times and the associated burden for patients.

Recently, Barrios et al. [8] introduced the cognitive fatigability assessment test (cFAST), a brief, 5 min tablet-based symbol digit modalities test (SDMT) variant designed to capture fatigability by measuring within-task performance change. Building on this approach, the present study directly compares fatigability indices from a long CPT and a short SDMT-based task, testing their ability to predict fatigue status and contrasting their diagnostic performance using ROC analyses.

Participants included 20 individuals with clinically confirmed MS (McDonald criteria) [9] and 20 healthy age-matched controls (HC). PwMS were relapse- and corticosteroid-free for at least 3 months and on stable disease-modifying therapy. HC reported no neurological or psychiatric disorders and scored ≤ 13 on the Beck Depression Inventory (BDI-II). Participants were excluded if they had current psychiatric conditions, relevant medication use, or recent neurological events. The study protocol was approved by the Ethics Committee of the University of Magdeburg and all participants gave written informed consent in accordance with the Declaration of Helsinki. An overview of sample characteristics is provided in Table 1.

**Table 1:** Group characteristics mean (± SD)

	HC	pwMS	
Gender [f/m]	11/9	15/5	*p* = .320
Age [years]	50.55 (15.90)	47.00 (13.40)	*p* = .323
BDI-II [points]	4.50 (3.80)	14.90 (6.98)	*p* < .001
BDI-FS [points]	1.00 (1.49)	3.75 (2.88)	*p* < .001
MFIS_total_ [points]	14.15 (10.99)	40.85 (12.10)	*p* < .001
PFS_cog_ [points]	6.60 (6.25)	17.85 (10.28)	*p* < .001
ESS [points]	7.30 (3.45)	11.20 (3.61)	*p* = .002
Disease duration [years]		13.50 (7.03)	
EDSS [points]		3.33 (1.81)	

*BDI-II* Becks depression inventory, *BDI-FS* Becks depression inventory fast-screen, *EDSS* Expanded disability status Scale, *ESS* Epworth sleepiness Scale, *HC* Healthy controls, *MFIS* Modified fatigue impact scale, *PFS* Pittsburgh fatigability Scale, *pwMS* People with multiple sclerosis

Objective fatigability was assessed with two computerized cognitive tasks (see Fig. 1): a brief, modified SDMT (mSDMT) and a prolonged CPT. The mSDMT was based on the cFAST [8] and a modified SDMT task from DeLuca et al. [10]. On each trial, participants judged whether a symbol–digit probe matched a reference key displayed on the screen. After a brief practice block (10 trials with feedback) and a 1 min calibration block to determine an individualized time limit, participants completed the 5 min experimental block under continuous time pressure, indicated by a shrinking on-screen bar. No feedback was given during the main block. The CPT [7] consisted of a sustained attention task presented in blocks of 5 min each. PwMS completed six blocks (~ 30 min total), whereas HC completed twelve blocks (~ 60 min total), reflecting the faster onset of fatigability typically observed in MS compared to healthy individuals. However, for comparability, analyses were restricted to the first six blocks in both groups. Trial procedures were otherwise identical between groups: on each trial, two grey bars were briefly presented, followed by a color change in one bar. Participants identified the color (red vs. blue) via button press while maintaining central fixation. Inter-trial intervals were jittered to increase attentional demands.

**Fig. 1:**
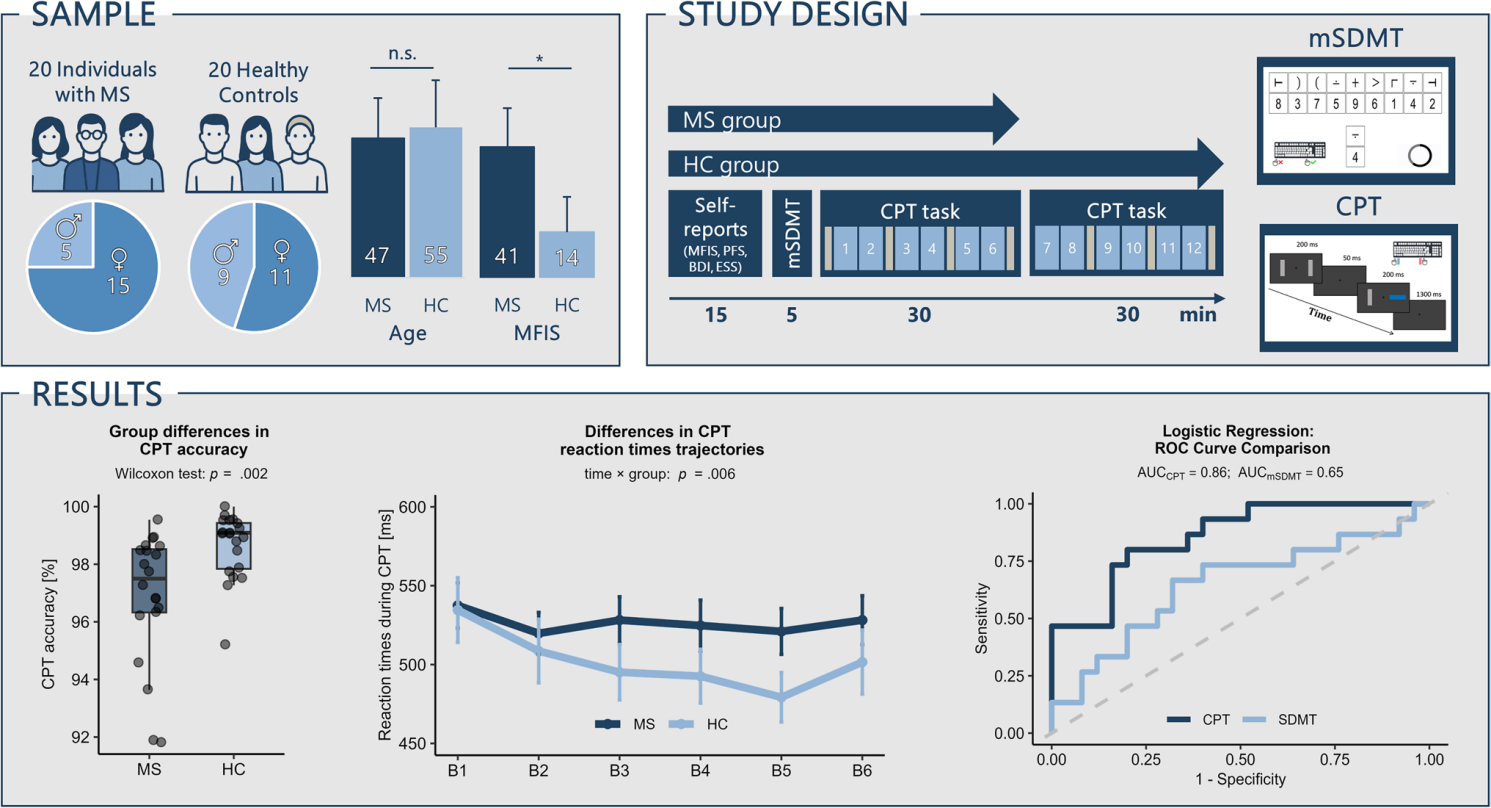
Sample, study design, and main results. Sample: characteristics of 20 people with multiple sclerosis (pwMS) and 20 age-matched healthy controls (HC). Groups were comparable in age and sex distribution, but pwMS reported significantly higher fatigue levels on the MFIS. Study design: following questionnaires, participants completed a brief 5 min modified symbol digit modalities test (mSDMT) and a continuous performance task (CPT; 30 min for MS, 60 min for HC). Schematic task displays illustrate the symbol–digit matching procedure of the mSDMT and the bar-color monitoring in the CPT, with participants responding via keyboard presses. Results: left: boxplot of group differences in CPT accuracy, showing reduced accuracy in MS group relative to HC. Two participants with MS showed lower accuracy values, but the group difference remained significant when these outliers were excluded. Middle: line plot of reaction time trajectories across CPT blocks, indicating that HC became faster over time, whereas MS group did not. Right: ROC curves comparing logistic regression models; CPT slopes yielded robust prediction of fatigue status, whereas mSDMT slopes showed poor predictive value

Before and after the mSDMT, and at regular intervals during the CPT, participants rated their momentary mental fitness and exhaustion using visual analogue scales (VAS). Questionnaires assessing fatigue (MFIS; Modified Fatigue Impact Scale), sleepiness (ESS; Epworth Sleepiness Scale), depressive symptoms (BDI-II), and perceived fatigability (PFS; Pittsburgh Fatigability Scale) [11] were also completed. Total testing time, including questionnaires and breaks, was approximately 90 min for pwMS and 120 min for HC (see Fig. 1).

To minimize the influence of extreme values, outliers in behavioral and self-report variables were identified separately within pwMS and HC and adjusted using winsorization (1.5 × interquartile range criterion). Mean behavioral performance (reaction time, accuracy, and coefficient of variation [COV]) and mean subjective VAS ratings were first compared between groups using *t* tests or Mann–Whitney *U* tests as appropriate. The COV, defined as the standard deviation divided by the mean reaction time, provided a normalized measure of intra-individual variability [12, 13]. Temporal changes (linear slopes) were analyzed separately for mSDMT and CPT using linear mixed-effects models with fixed effects of time, group, and their interaction, and random intercepts for participants. Logistic regression models were then fitted to predict fatigue status (MFIS > 38) [14] from task-derived slopes of reaction time, accuracy, COV, and VAS ratings of mental fitness and exhaustion for each cognitive task (mSDMT and CPT). Model performance was quantified using ROC analyses (AUC, sensitivity, specificity). Finally, multivariable logistic regression, including all slopes was conducted to assess combined predictive value, and variable importance was estimated using the *caret* package.

Both tasks were performed with high accuracy. In the mSDMT, group differences in accuracy and reaction time were non-significant, although pwMS showed a trend toward slower responses (*p* = 0.06). In the CPT, pwMS exhibited slightly lower accuracy than controls (*p* = 0.002; Fig. 1), while reaction times did not differ significantly between groups.

VAS ratings increased over time in both tasks, with consistently higher ratings in pwMS (all *ps* < 0.003). Only the CPT revealed differential behavioral trajectories: HC became faster over time, whereas pwMS did not (time × group, *p* = 0.006; Fig. 1).

Logistic regression models indicated that mSDMT slopes did not significantly predict fatigue status. The model showed limited discriminative ability (AUC = 0.65), particularly for detecting fatigued participants (sensitivity = 20%), suggesting limited diagnostic utility. In contrast, CPT slopes, particularly reaction time, COV, and VAS ratings on mental fitness predicted fatigue status (all *ps* < 0.05), yielding high discriminative accuracy (AUC = 0.86; sensitivity = 67%; specificity = 84%). ROC curve comparison suggested a trend toward higher AUC for CPT compared with mSDMT (DeLong’s test: *Z* = 1.85, *p* = 0.064; Fig. 1). Variable importance analysis confirmed that CPT reaction time, COV, and VAS ratings on mental fitness were the strongest predictors.

This study directly compared fatigability indices derived from a brief modified SDMT and a prolonged CPT in pwMS and HC. While subjective fatigability increased over time in both tasks, only the CPT revealed group-specific behavioral trajectories, with HC becoming faster over time, whereas pwMS did not. Crucially, logistic regression analyses demonstrated that CPT-derived slopes, particularly reaction time, COV, and VAS ratings on mental fitness, significantly predicted fatigue status. By contrast, mSDMT slopes failed to reach significance and showed limited discriminative capacity.

These findings align with prior work demonstrating that sustained attention paradigms, particularly CPT-like tasks, are sensitive to cognitive fatigability in MS [4, 6, 7]. Our results reinforce the idea that longer task durations provide more reliable behavioral markers by allowing fatigue-related decline to unfold, whereas brief tasks may fail to capture these effects robustly. In contrast, the short mSDMT variant provided insufficient diagnostic information. This is notable given that the task was conceptually inspired by the promising cFAST paradigm [8], which achieved good predictive validity in earlier work. Several task-related factors may explain this discrepancy. In cFAST, participants actively retrieve and enter digits, imposing higher cognitive-motor demands than the binary verification format used in our task. This difference likely reduced performance variability and attenuated fatigue effects. Moreover, differences in response modality (tablet vs. PC keyboard) and fatigability metrics (single ΔRT vs. multiple slopes) may further contribute to divergent outcomes. Together, these differences highlight that beyond task duration, factors such as response format, mode of administration, and metric operationalization critically shape fatigability detection in MS.

From a clinical perspective, these findings illustrate a clear trade-off between brevity and diagnostic utility. Although short tasks, like the mSDMT, are less burdensome for patients and more feasible in routine practice, their reduced predictive validity limits diagnostic utility. In contrast, CPT paradigms demonstrated robust discriminative performance, emphasizing the continued relevance of sustained attention paradigms for fatigue assessment in MS. Hybrid approaches that combine shorter behavioral tasks with physiological or smartphone-based markers [4, 15] may offer a promising compromise between feasibility and sensitivity.

Some limitations should be noted. The sample size, while comparable to prior fatigability studies, was modest and may limit generalizability. The reduced CPT duration for pwMS, implemented to mitigate patient burden, may have attenuated group differences relative to HC. However, only the first six blocks were included in the analyses for both groups, ensuring that the statistical comparisons were based on equivalent time spans. While persistent upper limb motor deficits in pwMS cannot be entirely ruled out, overall reaction times did not differ between groups, suggesting that general motor impairments did not systematically affect task performance. Fatigue classification was based on MFIS cutoffs, which, although widely used, remain subjective and may not fully capture the multidimensional nature of fatigue. Finally, BDI-II scores in the MS group were elevated compared with controls. However, none of the participants had a clinically diagnosed depression or were on antidepressant treatment, and the mean BDI-FS score (cognitive-affective subscale) was 3.75, below the commonly used clinical cutoff of 4. Importantly, re-running the logistic regression, including BDI-FS as a covariate confirmed that, while cognitive–affective depressive symptoms explained some additional variance, CPT slopes (reaction time and COV) remained significant predictors of fatigue status, indicating that cognitive fatigability captures aspects of MS-related fatigue beyond depressive symptoms.

In conclusion, in the present study, CPT-derived fatigability slopes reliably predicted fatigue status in pwMS, whereas the short mSDMT variant did not. These findings reinforce the continued relevance of sustained attention paradigms for objective fatigue assessment and highlight the challenges of developing brief yet sensitive alternatives for clinical use.

## Data Availability

Data sets are available from the corresponding author upon request.
